# Taxonomic Diversity of *Ranunculus* Section *Ranunculastrum* (Ranunculaceae) in Tajikistan, With an Identification Key and a New Species Based in Part on Molecular Phylogenetic Evidence

**DOI:** 10.1002/ece3.72191

**Published:** 2025-09-22

**Authors:** Andrey S. Erst, Mariyo T. Boboev, Komyor M. Boboev, Shukherdorj Baasanmunkh, Mathew T. Sharples, Alexander A. Kuznetsov, Maxim S. Kulikovskiy, Vera A. Kostikova, Svetlana Yu. Maltseva, Tatyana V. Erst, Mikhail V. Skaptsov, Kunli Xiang, Lian Lian, Hyeok Jae Choi, Wei Wang

**Affiliations:** ^1^ Central Siberian Botanical Garden Siberian Branch of Russian Academy of Sciences Novosibirsk Russia; ^2^ Kulob Botanical Garden Khatlon Science Center NAST Kulob Tajikistan; ^3^ Department of Biology and Microbiology Changwon National University Changwon South Korea; ^4^ Department of Biological and Earth Sciences Arkansas Tech University Russellville Arkansas USA; ^5^ Laboratory of the Herbarium Tomsk State University Tomsk Russia; ^6^ Laboratory of Molecular Systematics of Aquatic Plants К.А. Timiryazev Institute of Plant Physiology RAS, IPP RAS Moscow Russia; ^7^ Institute of Cytology and Genetics Siberian Branch, Russian Academy of Sciences Novosibirsk Russia; ^8^ Altai State University Barnaul Russia; ^9^ State Key Laboratory of Plant Diversity and Specialty Crops Institute of Botany, Chinese Academy of Sciences Beijing China

**Keywords:** endemic, phylogeny, Ranunculaceae, Ranunculastrum, *Ranunculus*, Tajikistan

## Abstract

*Ranunculus* L. (Ranunculaceae) is a substantial genus of flowering plants with worldwide representatives and marked centers of diversity in mountainous systems. One area of heightened species diversity in the genus lies in the Central Asian Mountains. Here, the taxonomic diversity of *Ranunculus* (sect. *Ranunculastrum* DC.) from the mountainous Central Asian nation of Tajikistan is investigated and discussed, with new identification keys for all species of this group provided. A heretofore unknown population of a distinct lineage of *Ranunculus* was recorded on the Darwaz Ridge of Tajikistan and found to be an undescribed species. Revision of herbarium materials (authentic, general collection), fieldwork, and newly obtained phylogenetic data pertaining to this and other taxa from this group allowed the description and illustration of a new, narrowly endemic species of the Pamir—*Ranunculus boboevii*. An assessment of the conservation status, a geographical distribution map, habitat circumscription, and an illustration for *R. boboevii* are provided. Taxonomic relationships, morphological differences, phylogenetic position, and endemism of *Ranunculus* in the context of the flora of Tajikistan are also discussed.

## Graphical abstract

Ranunculus section Ranunculastrum revision.

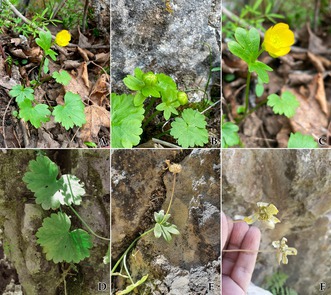

## 1 Introduction

*Ranunculus* L. is the largest genus in the buttercup family (Ranunculaceae) and is characterized by great morphological and geographic diversity, which is variously circumscribed by different authors (Tamura 1995). Approximately 600 species are distributed across all continents and most other landmasses, though the group is only poorly represented in the lowland tropics, and there mostly only as cosmopolitan weeds (Ziman and Keener 1989). Most species occur in temperate to arctic or subantarctic zones, especially in mountainous systems and often in relatively well‐watered habitats. Nevertheless, the genus is also considerably diverse in the Mediterranean region with ca. 160 species, with ca. 78 of those being endemic (Greatur et al. 1989; excluding microspecies of the apomictic *R. auricomus* complex). On other continents, several buttercups grow in regions with Mediterranean and otherwise subtropical climates (e.g., Cape region of South Africa, California, Florida, Central Chile) (Hörandl and Greilhuber 2002), and there are even taxa of relatively hot montane deserts that live well away from perennial water sources (Sharples, in prep.). The exact number of species‐rank and subspecific taxa is unknown due to the spontaneous emergence of new hybridogenous species and apomicts (Paun et al. 2006), as well as the poorly surveyed nature of many geographic regions, combined with the poorly surveyed nature of many herbarium collections—from both a morphological and a molecular perspective.

Despite much progress in resolving the phylogenetic relationships of many species of *Ranunculus* through molecular analysis (Hörandl and Emadzade 2012), many of the Asian members are still poorly investigated.

Recent morphological and genetic studies (Yang 2000; Erst 2007; Erst and Sukhorukov 2011; Almerekova et al. 2020; Erkul et al. 2021; Sinan et al. 2021) have revealed expanded taxonomic diversity within selected complexes of Asian *Ranunculus*. More than 90 species of *Ranunculus* are distributed across Central Asia (Kovalevskaya 1972), and 41 have been recorded in Tajikistan according to the most recent data (Ovchinnikov 1975). Differentiation in the genus and formation of locally endemic species in the region are closely related to the uplift and glaciation history of the Tian Shan and Pamir‐Alay Mountains (Shchegoleva et al. 2022).

Section *Ranunculastrum* DC. of the genus *Ranunculus* includes ca. 70 species natively distributed in mostly Mediterranean climates of southern Europe, northern Africa, southwestern Asia, Central Asia, and northwestern China and Nepal (Kadota 1991; Tamura 1995). The section is characterized by tuberous or torulose roots with fibrous secondaries, as well as slightly to strongly compressed achenes. This section is quite heterogeneous in appearance, and the fruits are especially variable and exhibit sometimes quite elaborate structures. Some species are stoloniferous, while others are not able to spread via horizontal aboveground shoots (Tamura 1995). Based on combined morphological and molecular data, this section is currently paraphyletic but does not include another section name described before (Hörandl and Emadzade 2012). Species in this group occur from rather arid, grassy, gravelly to desert habitats; to the edges of scrub and forest margins, sometimes in pastures as a weed; and in general, from lowlands up to the alpine zone, including adjacent to snowfields (Tamura 1995).

During botanical fieldwork in Tajikistan in early spring of 2022, a new population of an unfamiliar *Ranunculus* was recorded on the Darwaz Ridge in the Pamir. Further work based upon comparison of herbarium material and the taxonomic literature allowed us to describe this species as new to science. The search for further localities of the new taxon was not successful, which suggests it is a rare and very locally endemic buttercup. Local endemism is a common feature of the flora of Tajikistan, which shelters over 1400 endemic species, steadily increasing in number (Nowak et al. 2022).

## 2 Materials and Methods

A study of herbarium material to produce a dichotomous key to Section *Ranunculastrum* in Tajikistan and to confirm the status of the new population as a distinct species was undertaken at the following collections: ALTB, BM, E, K, LE, MW, NS, NSK, TAD, TASH, TK, and the B. Gafuvor Herbarium at Khujand State University (Thiers 2017). An illustration of the new species of *Ranunculus* was based upon its type specimens as well as upon field insights (Erst A.S. & Boboev M.T.: TJ‐2022‐16). Museum collection records were further employed to compile a distribution map for this species using Arc‐GIS (Esri 2018), and the species' extent of occurrence (EOO) and the area of occupancy (AOO) were estimated using GeoCAT‐Kew (Bachman et al. 2011), based on these records as well as field observations. For the distribution map and for estimation of EOO and AOO, we included specimens examined in the previously referenced studies, using approximate georeferenced coordinates following data in the species protologue. The conservation status of the new species was assessed according to the categories and criteria of the IUCN (2022).

### 2.1 Phylogenetic Studies

#### 2.1.1 Taxon Sampling

We sampled 114 individuals deriving from 112 species of *Ranunculus*, covering all nine major clades of the genus and including 26 species occurring in Tajikistan (Hörandl and Emadzade 2012). We also sampled *Ceratocephala*, *Krapfia*, and *Myosurus* in Ranunculeae to represent outgroups (Table S1).

#### 2.1.2 DNA Extraction, Amplification, and Sequencing

DNA isolation was conducted using a Plant Genomic DNA Kit (TIENGEN Biotech, Beijing, China) according to the manufacturer's protocol. The nuclear DNA region ITS (ITS1, 5.8S rRNA, and ITS2) and plastid DNA region *mat*K were amplified by means of a thermal cycler (BioRad) using the 2X PCR Taq Plus MasterMix with dye (Applied Biological Materials Inc., Canada). Primers used in this study are listed in the Table S2.

To obtain sequences for the regions of interest, PCR amplification was carried out according to the following parameters. For ITS, initial denaturation was set for 3 min at 94°C, followed by 35 amplification cycles with 30 s at 94°C, 30 s at 50°C–54°C, 1 min at 72°C, and elongation for 7 min at 72°C. For *mat*K, initial denaturation was set for 3 min at 94°C, followed by 35 amplification cycles with 30 s at 94°C, 1 min at 51°C, 1 min at 72°C, and the final extension phase for 10 min at 72°C.

#### 2.1.3 Molecular Methods

Sequences were aligned using Geneious 10.1.3 (Kearse et al. 2012) and adjusted manually. Phylogenetic reconstruction was first conducted separately for nuclear and plastid data. No topological incongruence with a high support value (posterior probabilities and bootstrap percentages) was found. Therefore, the nuclear and plastid sequences were combined into one dataset for phylogenetic analyses using Geneious 10.1.3 (Kearse et al. 2012). Phylogenetic trees were reconstructed using Maximum Likelihood (ML) and Bayesian Inference (BI). We used Partition Finder v2.1.1 (Lanfear et al. 2017) to simultaneously infer both the best‐fitting nucleotide substitution models and the partitioning scheme. For ML, we employed RAxML 8.0 (Stamatakis 2014) with 1000 bootstrap replicates. For BI analyses, two independent analyses consisting of four Markov Chain Monte Carlo (MCMC) chains were run, sampling one tree per 1000 generations for 20 million generations. Runs were completed when the average standard deviation of split frequencies reached 0.01. The stationarity of the runs was assessed using Tracer v1.6 (Rambaut and Drummond 2014). After removing the burn‐in period samples (the first 25% of sampled trees), a majority rule (> 50%) consensus tree was constructed. Nodes with bootstrap support (BS) ≥ 70% (Hillis and Bull 1993) and PP ≥ 0.95 (Ronquist and Huelsenbeck 2003) were regarded as being well supported. Trees were visualized using FigTree v.1.4.0 (Rambaut 2012).

## 3 Results

### 3.1 Molecular Data

**FIGURE 1:**
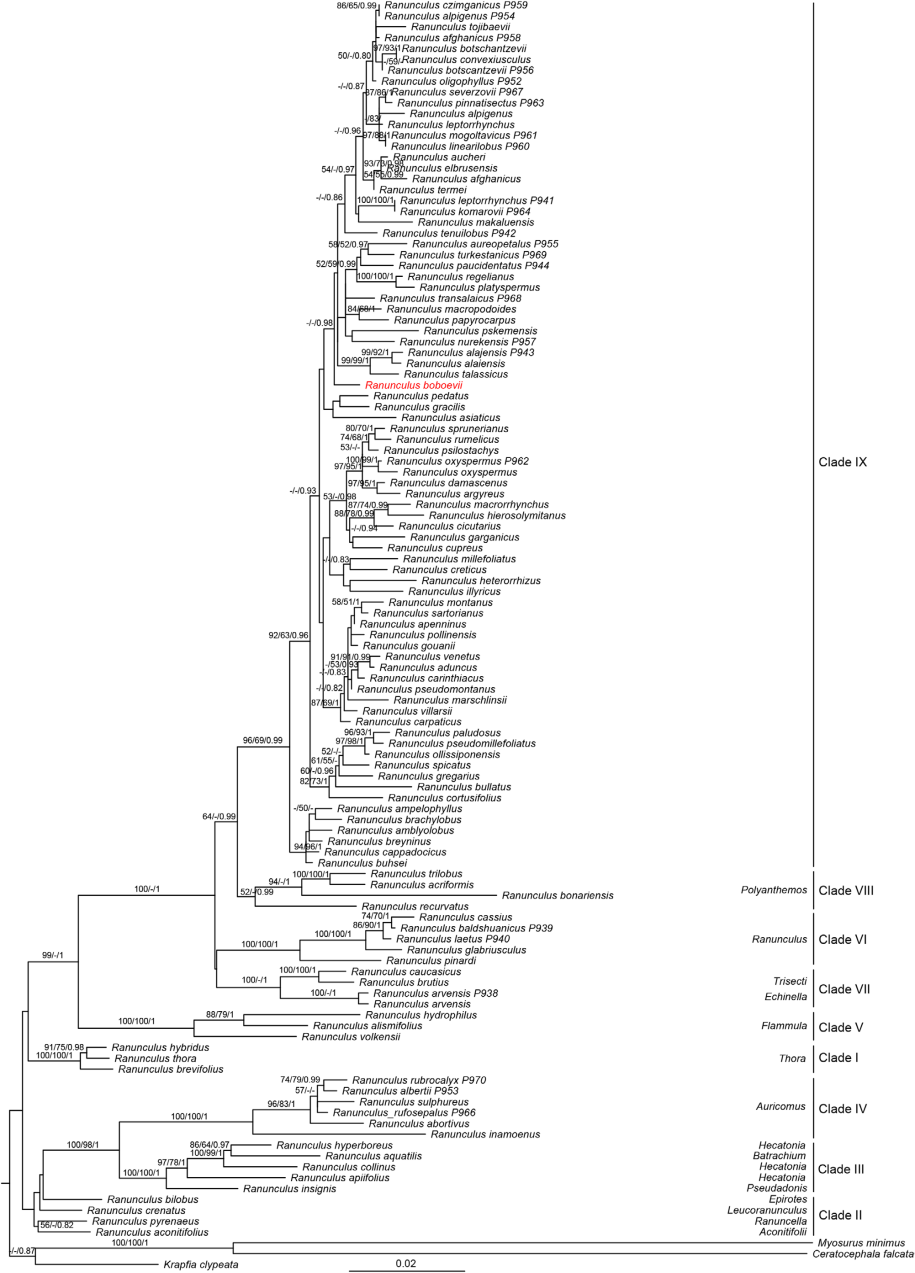
Maximum likelihood (ML) phylogenetic tree of *Ranunculus* species and three outgroup taxa constructed using nuclear and plastid sequences. Numbers above branches are bootstrap percentages (ML/MP) and Bayesian posterior probabilities, respectively. Only support values of BS ≥ 50% and PP ≥ 0.80 are shown. The classification is given according to Hörandl and Emadzade (2012).

Final alignments contained 95 taxa with 632 bp for ITS and 1871 bp for *mat*K. BI analyses resulted in identical trees that were highly consistent with the tree derived from the ML analysis (Figure 1). In *Ranunculus*, Clade IX is monophyletic with medium to strong support (BS = 96% and PP = 0.99). All of the 26 sampled species of Tajikistan were nested in Clade IX. The new species of *Ranunculus* was thus reconstructed as a member of Clade IX and was found sister to the *Ranunculus czimganicus–Ranunculus talassicus* clade with good statistical support (PP = 0.98).

### 3.2 Taxonomic Treatment

**FIGURE 2:**
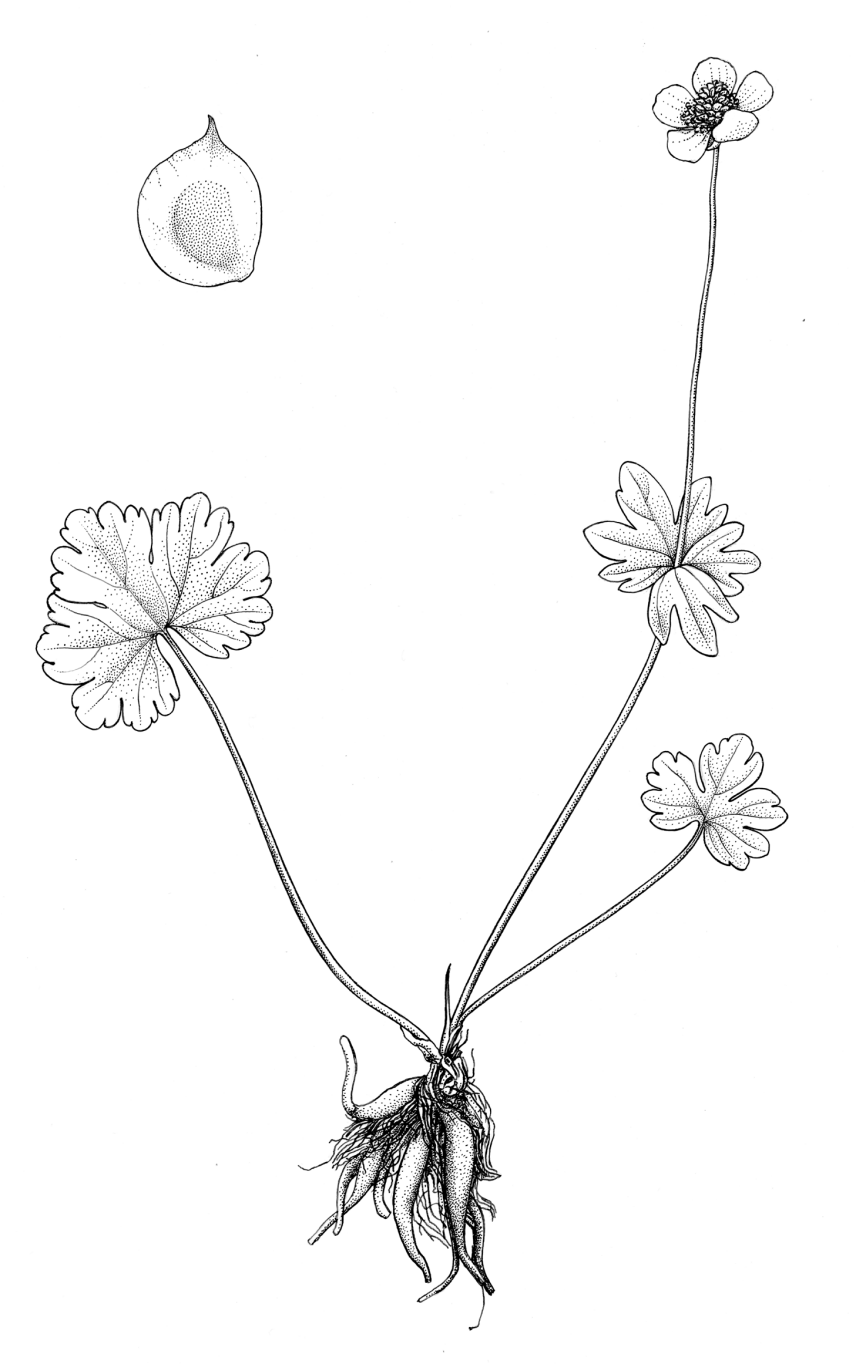
General view of *Ranunculus boboevii* (depicted by Natalia Prydak).

**FIGURE 3:**
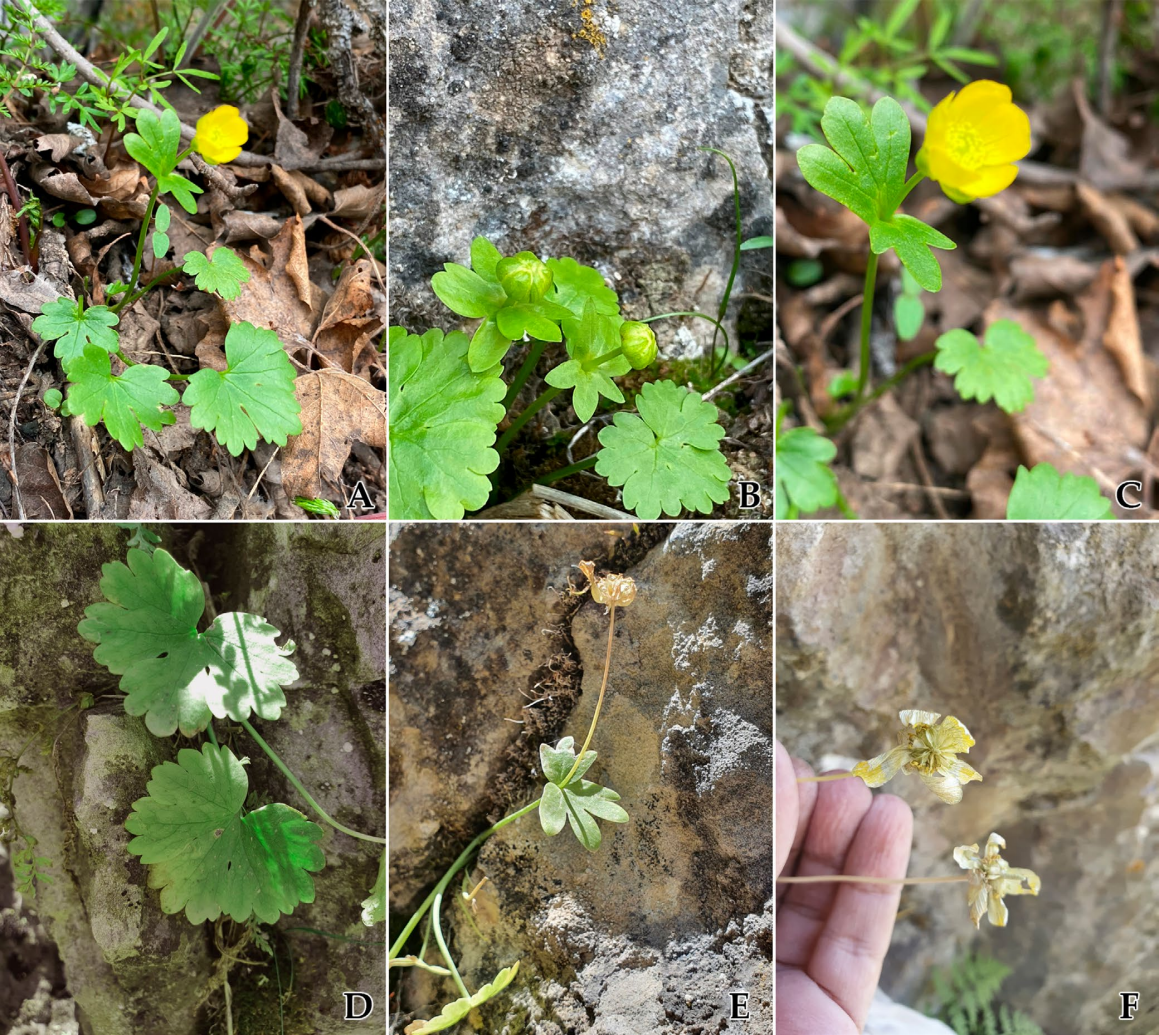
Wild photographs of *Ranunculus boboevii*. (A) General view in flowering time, (B) Basal leaf in flowering time, (C) Cauline leaf in flowering time, (D) Basal leaf in fruiting time, (E) In fruiting time, (C) Flowers and seeds in the beginning fruiting time (depicted by Andrey Erst and Mariyo Boboev).

*Ranunculus boboevii* Erst & Boboev, sp. nov. [urn:lsid:ipni.org:names: 77368948‐1] (Figures 2, 3).

#### 3.2.1 Type

Tajikistan. Sh. Shohin Raion, Darwaz Ridge, basin of Obiniob River, around Kalay‐Khuhna old Village, 38.009080N, 70.259786E, 2304 m a.s.l., 06 May 2022, Erst A.S., Bovoev M.T., TJ‐2022‐16 (holotype: NS‐0000933; isotypes: NS‐0000934, NS‐0000935, NS‐0000936, NS‐0000937, NS‐0000938, NS‐0000939); Paratype: Tajikistan. Sh. Shohin Raion, Darwaz Ridge, basin of Obiniob River, around Kalay‐Khuhna old Village, 38.009080N, 70.259786E, 2304 m a.s.l., 06 Apr. 2023, Bovoev M.T., TJ‐2023‐1 (NS‐0000940).

#### 3.2.2 Diagnosis

*Ranunculus boboevii* is similar to *R. mindshelkensis* and *R. aureopetalus* morphologically but differs in its height, thin stem texture, glabrous stems, reniform‐suborbicular basal leaf blades, maximum dissection of the basal leaves, thin leaf texture, cauline leaf width, number of lobes and segments of the cauline leaves, leaf lobe width, lanceolate receptacle, seed length and width, straight stylodium (beak), and habitat in thermophilous forest and shrubland (Table 1).

**TABLE 1:** Morphological differences between *Ranunculus boboevii* and closely related species.

Characters	*Ranunculus boboevii*	*Ranunculus mindshelkensis*	*Ranunculus aureopetalus*
Plant height (cm)	12–27	5–6 (15–25)	7–10 (15–25)
Number of flowers in the inflorescence	1–2	1–4	1–3
Stem position	Slender or suberect	Erect	Slender or erect
Stem texture	Thin	Thick	Thick
Stem pubescence	Glabrous	Sparsely pubescent or glabrous	Sparsely pubescent or glabrous
Basal leaf blade length (cm)	2–4	1–2	1.5–3.2
Basal leaf blade width (cm)	3–7	2–2.5	2.5–4 (5)
Basal leaf petiole length (cm)	7.5–17	1.5–3	3–8
Basal leaf shape	Reniform‐suborbicular	Reniform‐rounded	Orbicular‐cordate
Maximum dissection of the basal leaf (cm)	1–2	0.7–1	0.6–1.5
Number of lobes of the basal leaf	3	3	3–5
Number of teeth in the central lobe of the basal leaf	3–9	3–5	7–12
Basal leaf texture	Thin	Thick	Thick
Basal leaf pubescence	Glabrous	Glabrous	Glabrous
Cauline leaves length (cm)	0.5–3.5	0.5–1	1.1–2.4
Cauline leaf blade width (cm)	3–4	2–3	1.1–1.9
Maximum dissection of the cauline leaf (cm)	0.5–2	0.3	0.9–1.9
Petiole of cauline leaf presence	Absent	Absent	Absent
Number of segments of the cauline leaf	1–2	1	1
Number of lobes of the cauline leaf	3–4	3	3
Lobe width (cm)	0.5–1	0.1–0.3	0.2–0.4
Number of teeth in the central segment of the cauline leaf	1–9	3	1–5
Flowers diameter	1.5–3	1–1.5	2–3
Sepal length (cm)	0.5–1.2	0.5–0.6	0.8–1
Sepal width (cm)	0.2–0.5	0.2–0.3	0.3–0.4
Sepal pubescence	Glabrous	Glabrous	Glabrous
Petals length (cm)	0.4–1.2	0.8–1	1–1.5 (1.8)
Petals width (mm)	0.3–0.8	0.3–0.5	0.5–0.9
Anther length (mm)	0.2–0.3	0.1–0.2	0.2–0.3
Receptacle shape	Lanceolate	Narrow cylindrical	Cylindrical
Receptacle pubescence	Glabrous	Glabrous	Glabrous
Seeds length (cm)	0.3–0.5	0.5–0.7	0.2–0.3
Seeds width (mm)	0.3–0.4	0.4–0.7	0.2–0.3
Beak shape	Straight	Hamate curved	Curved
Beak length (cm)	0.1	0.3–0.4	0.2–0.3
Ecology	Thermophilous forests and shrublands	Alpine grasslands and pastures	Juniper woods and scrub

#### 3.2.3 Description

*Slender*, *creeping to suberect perennial herb*, stems 12–27 cm tall, glabrous, thin in texture, shoots simple or two‐branched, roots dimorphic, some fibrous and oblong‐fusiform, others tuberous, 1.2–5 cm long. *Basal leaves* reniform‐suborbicular to somewhat divided 1/2–1/3 to the base into 3 broadly cuneate, obtuse, to crenate, overlapping lobes, with 3–9 teeth in the central lobe; petioles 7.5–17 cm long; blades 2–4 cm long, 3–7 cm wide, thin in texture, glabrous on both sides. *Cauline leaves* are smaller, 1–2‐dissected, with segments 3–4‐partite, broadened, lobes apically rounded, 0.5–1 cm wide, with 1–9 teeth on the central lobe; petioles are absent; blades are 0.5–3.5 cm long, 3–4 cm wide, thin in texture, and glabrous on both sides. *Inflorescence* solitary to 2‐flowered. *Flowers are* 1.5–3 cm in diameter. *Sepals* 5, reflexed at anthesis, ovate‐concave, 0.5–1.2 cm long, 0.2–0.5 mm wide, with a membranous margin up to 0.5 mm wide, glabrous. *Petals* yellow, ovate, 0.4–1.2 cm long, 0.3–0.8 cm wide, glabrous, apex obtuse. *Anthers* are 0.2–0.3 cm long. *Aggregate fruiting heads* subglobose to broadly ovate. *Receptacle* lanceolate, glabrous. *Achenes* 0.3–0.5 cm long (including beak) by 0.3–0.4 cm wide, body suborbicular, flat, broadly winged, glabrous, beak 0.1–0.2 cm long, straight.

#### 3.2.4 Phenology

Flowering in April and May. Fruiting in May and June.

#### 3.2.5 Distribution

**FIGURE 4:**
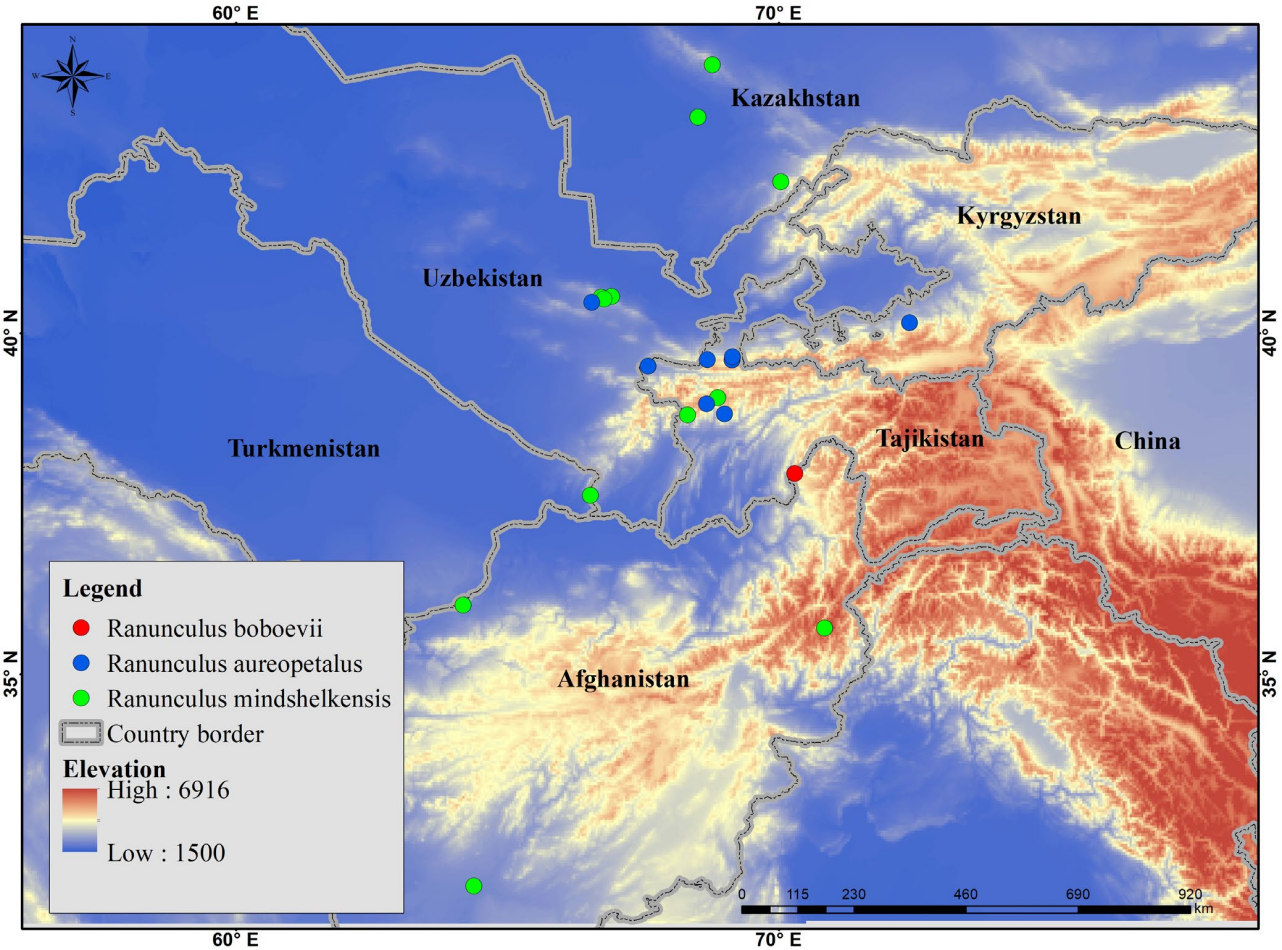
Distribution map of *Ranunculus boboevii* (red), *R. aureopetalus* (blue), and *R. mindshelkensis* (green). Museum collection records were further employed to compile a distribution map for this species using Arc‐GIS [19], and the species' extent of occurrence (EOO) and the area of occupancy (AOO) were estimated using GeoCAT‐Kew (Bachman et al. 2011), based on these records as well as field observations.

*R. boboevii* is a local endemic of Darwaz Ridge in the Pamir Mountains. The closely related *R. mindshelkensis* is endemic to Middle Asia (Tajikistan, Uzbekistan, Turkmenistan, Kazakhstan, and Afghanistan), and *R. aureopetalus* is endemic to the Pamir‐Alay (Figure 4).

#### 3.2.6 Habitat and Plant Associations

*R. boboevii* grows in crevices and stony soil pockets of steep schistose rock, not directly in the ground (Figure 4). Community associates include *Juniperus seravschanica* Kom., *Malus sieversii* (Ledeb.) M. Roem., *Amygdalus bucharica* Korsh., *Acer turkestanicum* Pax, *Lonicera zaravschanica* Pojark., *Cystopteris fragilis* (L.) Bernh., *Cheilanthes persica* (Bory) Mett. ex Kuhn, *Dictamnus tadshikorum* Vved., *Prangos pabularia* Lindl., *Fritillaria bucharica* Regel, *Fritillaria eduardii* A. Regel ex Regel, *Eremurus robustus* (Regel) Regel, *Eremurus olgae* Regel, *Eremurus korshinskyi* O.Fedtsch., *Vicia tenuifolia* Roth, *Scutellaria zaprjagaevii* Kochk. & Zhogoleva, *Tulipa praestans* H.B.May, *Iris darwasica* Regel, *Juno bucharica* (M.Foster) Vved., *Ostrowskia magnifica* Regel, *Poa bulbosa* L., *Nepeta podostachys* Benth., *Lophanthus ouroumitanensis* (Franch.) Kochk. & Zuckerw., *Rheum maximowiczii* Losinsk., *Polygonatum sewerzowii* Regel, *Impatiens parvifora* DC., *Adonis aestivalis* L., *Anemone bucharica* (Regel) Finet & Gagnep., *Anemone tschernaewii* (Czern.) Regel, *Thalictrum kuhistanicum* Ovcz. & Kochk., *Rosularia hissarica* Boriss., and *Arctium lappa* L. Epilithic mosses and lichens are also common associates.

#### 3.2.7 Conservation Status

*R. boboevii* is an extremely narrowly distributed endemic species of the Pamir Mountains of Tajikistan, to date known from one population of two or three individuals per m^2^ within an area of < 500 m^2^. It should therefore be assigned the status EN (Endangered) under Criteria B1 ab (i, ii, iii) + B2 ab (i, ii, iii) following the IUCN Standards and Petitions Committee (IUCN 2022). Because of its relatively inaccessible habitats, it is not thought to be under immediate terminal threat from grazing, development, and other disturbance pressures.

#### 3.2.8 Etymology

*R. boboevii* is named after Tillo Boboev of Tajikistan, the founding director of the Kulob Botanical Garden. Tillo Boboev served as professor at the Academy of Sciences of Tajikistan and actively promoted botanical science in Central Asia.

## 4 Key to *R. boboevii* and Species of Sect. *Ranunculastrum* From Tajikistan

**Table:** 

1	Achenes with pendulous appendages at the base, densely fused with receptacles, beaks sharp, and rigid	*R. oxyspermus*
+	Achenes lacking appendages at the base, weakly attached to the receptacle and easily detaching when ripe, beaks soft, and not rigid	2
2	Leaves only basal, 5–9 dissected into deeply and unevenly divided segments. Receptacle and achenes glabrous, beak black. Stoloniferous alpine plants with numerous daughter plants	*R. turkestanicus*
+	Cauline leaves present; these are sometimes reduced	3
3	Leaves entire, dentate, lobed, or not deeply broadly 3‐dissected, elliptic to rounded	4
+	Leaves more complex, deeply divided or dissected or tripartite almost to the base, typically with deeply twice‐dissected lateral segments	9
4	Leaves elliptic or broadly cuneate, leaf apex truncate, 2–3‐toothed or 3‐lobed	5
+	Leaves reniform‐rounded or orbicular‐cordate	6
5	Plant densely white pubescent. Fruits narrowly winged, pubescent, up to 3 mm long	*R. paucidentatus*
+	Plant glabrous, stem sometimes slightly pubescent. Fruits cariniform, glabrous, 2–3 mm long	*R. alajensis*
6	Basal leaves with a broad or narrow base, reniform. Achenes pubescent	7
+	Basal leaves with straight, rounded, or cuneate bases. Fruits pubescent or glabrous	8
7	Leaves orbicular‐cordate, with a narrow sinuate base, not coriaceous. Peduncles are flexuous, elongated during fruiting. Cauline leaves sessile, elongated, and 2–3 times dissected into linear to lanceolate 3‐toothed lobes	*R. chaffanjonii*
+	Leaves reniform‐cordate, with a broad sinuate base, more or less coriaceous. Peduncles straight or almost straight. Cauline leaf lobes entire	*R. olgae*
8	Leaves with cuneate or truncated bases. Fruits are hairy. Plants are not less than 15–30 cm high	*R. czimganicus*
+	Leaves with a cuneate base. Fruits glabrous. Plants (3) 5–6 cm high	*R. mindshelkensis*
9	Stems appressed pubescent. Leaves 1–2‐pinnatisected or 2–3‐trisected, with elongated linear lobes	10
+	Stems, at least in the lower half, protruding, protruding‐pubescent, or glabrous. Leaf segments and lobes are wider	11
10	Leaves 2–3‐trisected; segments or segment lobes elongate, linear. Achenes glabrous	*R. linearilobus*
	Leaves 1–2‐pinnatisected, with short serrate terminal lobes. Fruits finely pubescent	*R. pinnatisectus*
11	Calyx and stem glabrous, the peduncles sometimes pubescent	0.12
+	Calyx, peduncles, and often stem‐pubescent	14
12	Flowers solitary or several in an inflorescence, 1.5–3 cm in diameter. Leaves orbicular‐cordate, deeply triquinate with large crenate‐lobed segments	13
+	Flowers solitary, 1.2–1.4 cm in diameter. Leaves cordate‐reniform, trisected to base	*R. jazgulemicus*
13	Stems and leaves thin, glabrous. Basal leaf blade reniform‐suborbicular, petiole 7.5–17 cm, cauline leaf blade 3–4 cm wide, the lobes wide (0.5–1 cm). Achenes 0.3–0.5 cm long, 0.3–0.4 cm wide, beak straight, 0.1 cm long	*R. boboevii*
+	Stems and leaves thick, glabrous, or sparsely pubescent. Basal leaf blade orbicular‐cordate, petiole 3–8 cm; cauline leaf blade 1.1–1.9 cm wide, the lobes narrow (0.2–0.4 cm). Achenes 0.2–0.3 cm long, 0.2–0.3 cm wide, beak curved, 0.2–0.3 cm long	*R. aureopetalus*
14	Leaves 3–4 ternate‐dissected; segments petiolate, with narrow thin lobes	15
+	Leaves 1–2 ternate‐partite or 3–5 dissected	17
15	Achenes 2.5–3 mm long, slightly convex, sparsely pubescent, beak 0.8–1 mm long, arranged in small rounded heads 0.8–1 cm long	*R. tenuilobus*
+	Achenes (3) 4–6 mm long, flat, slightly pubescent or glabrous, with the beak longer, and fruits arranged in rounded or oblong larger heads	16
16	Fruits 3–4.5 mm long, with cilia, mainly along the ventral margin. Fruit head oblong‐cylindrical. Flowers are 1.5–2 cm in diameter, more or less numerous	*R. komarovii*
+	Fruits 5–6 mm long, glabrous. Fruit head rounded. Flowers 2.5–3 cm in diameter, solitary	*R. regelianus*
17	Leaves 3–5 dissected to base or almost to base	18
+	Leaves trisected	0.19
18	Achenes pubescent, compressed laterally. Leaves, and typically stems, glabrous	*R. botschantzevii*
+	Achenes glabrous, almost flat. Stems and leaves pubescent	*R. transalaicus*
19	Leaves 3–4 trisected. Achenes pubescent	20
+	Leaves 1–2 trisected into wider 2–3‐partite and sometimes dissected segments; lateral segments typically sessile. Achenes glabrous or sparsely hairy on the sides	21
20	Flowers 2.5–2.8 cm in diameter. Petals broadly ovate. Roots brownish‐gray	*R. badachschanicus*
+	Flowers not more than 2–2.2 cm in diameter. Petals oblong‐ovate. Roots thickened whitish	*R. alpigenus*.
21	Achenes with sparse, long cilia along the edge. Leaf segments are broad, more or less obovate, rhombic, and deeply tripartite into 2–3 incised, slightly serrate, or entire lobes. The fruit head is oblong‐elliptical or more or less rounded	*R. leptorrhynchus*
+	Achenes glabrous	22
22	Leaves trisected, segments long, more or less deeply three‐lobed. Flowers are 2.5–3 cm in diameter. Fruit heads up to 2.2 cm long. Achenes 7–7.5 mm long, with a 3–3.5 mm long beak, curved at the apex. Stems with long, sometimes drooping trichomes	*R. severtzovii*
+	Leaves smaller, leaflets 1–2 trisected. Flowers are 1.7–2 cm in diameter. Fruit head 8–9 mm long. Achenes 4–5 mm long, with a thin, 2–2.5 mm long beak. Stems pubescent distally	*R. mogoltavicus*.

## 5 Discussion

Tajikistan is home to one of the world's richest temperate floras and harbors up to 4500 vascular plants (Nowak et al. 2020). A large proportion of them (more than 30%) are endemic to Tajikistan (Nowak et al. 2020, 2022). The flora of this region has diversified across numerous ecological niches, as the region is characterized by both massive mountain systems with deep canyons and cooler climates and lowland valleys with nearly subtropical climates. The Pamir‐Alay mountains limit plant migration and have provided conditions for independent diversification of non‐sister groups in isolation, which have speciated at different times across this exceptional elevational gradient.

In the flora of Tajikistan, the genus *Ranunculus* is represented by various groups, both mesophytic and xerophytic. The section *Ranunculastrum* is characterized by the greatest species diversity within Tajik buttercups, as well as a large number of endemic taxa, including 23 species that are restricted to desert, steppe, foothill, montane, or alpine habitats (Ovchinnikov 1975). A high level of endemism in this group is likely due to autochthonous diversification in the mountains of Central Asia, stemming from regional adaptive diversification of its ancient Mediterranean predecessors (Shchegoleva et al. 2022).

Our phylogenetic reconstruction produced similar results with previous work (Hörandl and Emadzade 2012). In *Ranunculus*, most species of Tajikistan are members of the section *Ranunculastrum*, including the new local endemic species *R. boboevii* from Pamir (Figure 1). Section *Ranunculastrum* is quite heterogeneous in appearance, especially in its aforementioned variable achene architecture (Tamura 1995). The studied group is paraphyletic but is nonetheless characterized by beaks mostly equaling or exceeding the length of the achene body; these borne on an often long or short triangular, entirely glabrous receptacle, with partly tuberous roots and mostly elongated aggregate fruits (Hörandl and Emadzade 2012). In the flora of Tajikistan, the representatives of section *Xiphocoma* (Stev.) Ovcz. and *Pterocarpa* Ovcz. are included within section *Ranunculastrum* (Tamura 1995). The first group is represented by one species (*R. oxyspermus* Bieb.), which differs from the species of the second group in its achenes with a pendulous appendage at the base, which are densely fused with the receptacle and bent downwards (vs. fruits weakly attached to the receptacle, easily detaching when ripe). Thus, while *Ranunculastrum* s.str. as such may well form a natural group in a wider context, this is not necessarily true for sect. *Xiphocoma*. Even accepting the treatment as proposed, the current taxonomic situation remains, at best, inconclusive as regards the less derived species, viz., sect. *Pterocarpa* (Goepfert 1976).

The assignment of species from Tajikistan to more accurate taxonomic groups requires additional data, including new sequences for species of unclear affinity or local endemics, for example, *Ranunculus oligophyllus* Pissiauk and *R. chodzhamastonicus* Ovcz. & Junussov. We now consider the latest work on the classification of the genus *Ranunculus* and assign the studied species to the paraphyletic section *Ranunculastrum* (Hörandl and Emadzade 2011).

Representatives of Section *Ranunculastrum* from Tajikistan are characterized by leafless ( *R. turkestanicus* ) or leafy stems (other species). Other species of the group differ mainly in the morphological structure of leaves. Some species have entire, serrate, lobed, or not deeply tripartite leaves that are elliptical (*R. alaensis*, *R. paucidentatus*) or rounded (e.g., *R. minddshelkensis*). This group includes species with rigid erect stems and thick leaf texture (*R. olgae*, *R. chaffajonii*) or species with fine leaf texture and nodding shoots (*R. boboevii*, *R. jazgulemicus*). The species of this group also differ in the pubescence level of peduncles and achenes. The second large group comprises species with deeply partite, pinnately dissected leaves (*R. linearilobus*, *R. tenuilobus*, etc.). Within the group, the species differ in the dissection of the basal leaf blade and the pubescence of the peduncles, calyx, and fruits.

According to morphological and molecular data, *R. boboevii* belongs to clade IX, sect. *Ranunculastrum* s.str. The new species, along with *R. pedatus* , *R. asiaticus* , and *R. gracilis* , represents the basal sampled members of this clade as inferred through their sister status to the rest of the molecular clade (Figure 1). *R. boboevii* is morphologically similar to *R. mindshelkensis* (not included in the molecular analysis due to lack of fresh specimens) and *R. aureopetalus*. The new species occupies a separate phylogenetic position from its morphologically similar species and differs greatly in the peduncle pubescence and especially in the structure of cauline leaves with broad and poorly dissected segments and lobes (Figure 3B). Basal leaves are morphologically similar to those in the endemic *Ranunculus veronicae* from sect. *Ranunculastrum* (not included in the molecular analysis) was recently described from western Crete (Greece) (Böhling 2000), but the two species probably show only distant affinity, as this feature was likely formed due to parallel evolution under similar growing conditions in stony substrates, given the biogeographic distance involved.

Despite long‐term botanical exploration in Tajikistan and the presence of numerous herbarium collections valuable for systematic and other investigations, herbaria and GenBank (NCBI) generally lack modern, fresh specimens or sequences (respectively) that are optimal for use in molecular analysis. A number of papers have recently addressed the study of individual representatives of the genus *Ranunculus* and the description of new species from Central Asia, including those from Tajikistan (Shchegoleva et al. 2020, 2022, etc.), but the genus is, in general, still not well elucidated, and new taxa will continue to be located.

## Author Contributions

**Andrey S. Erst:** conceptualization (equal), data curation (equal), formal analysis (equal), funding acquisition (equal), investigation (equal), methodology (equal), project administration (equal), resources (equal), software (equal), supervision (equal), validation (equal), visualization (equal), writing – original draft (equal), writing – review and editing (equal). **Mariyo T. Boboev:** resources (equal), writing – review and editing (equal). **Komyor M. Boboev:** investigation (equal), resources (equal), writing – review and editing (equal). **Shukherdorj Baasanmunkh:** conceptualization (equal), investigation (equal), resources (equal), writing – review and editing (equal). **Mathew T. Sharples:** conceptualization (equal), data curation (equal), methodology (equal), writing – original draft (equal), writing – review and editing (equal). **Alexander A. Kuznetsov:** conceptualization (equal), data curation (equal), resources (equal). **Maxim S. Kulikovskiy:** conceptualization (equal), data curation (equal), software (equal). **Vera A. Kostikova:** conceptualization (equal), data curation (equal), methodology (equal), validation (equal), writing – review and editing (equal). **Svetlana Yu. Maltseva:** validation (equal), visualization (equal), writing – review and editing (equal). **Tatyana V. Erst:** conceptualization (equal), data curation (equal), resources (equal), visualization (equal), writing – review and editing (equal). **Mikhail V. Skaptsov:** investigation (equal), validation (equal), writing – review and editing (equal). **Kunli Xiang:** conceptualization (equal), data curation (equal), formal analysis (equal), funding acquisition (equal), software (equal), visualization (equal), writing – review and editing (equal). **Lian Lian:** resources (equal), software (equal), writing – review and editing (equal). **Hyeok Jae Choi:** conceptualization (equal), formal analysis (equal), funding acquisition (equal), resources (equal), software (equal), validation (equal). **Wei Wang:** data curation (equal), formal analysis (equal), funding acquisition (equal), project administration (equal), software (equal), supervision (equal), writing – review and editing (equal).

## Conflicts of Interest

The authors declare no conflicts of interest.

## Supporting information


**Table S1:** Accession numbers of samples used for phylogenetic analyses of *Ranunculus*.

**Table S2:** Primers used for amplification and sequencing in this study.

## Data Availability

The DNA sequences generated in the present study have been deposited in the National Center for Biotechnology Information (NCBI) database. The accession numbers and the information on the voucher specimens are available in Table S1. The voucher specimens of the new species were housed in NS.
