# Supplementary material for: Taxonomic Diversity of Ranunculus Section Ranunculastrum (Ranunculaceae) in Tajikistan, With an Identification Key and a New Species Based in Part on Molecular Phylogenetic Evidence
Source: Ecol Evol. 2025 Sep 22;15(9):e72191. doi: 10.1002/ece3.72191 (PMC12453610; doi:10.1002/ece3.72191)
Supplement: Supplementary file 1 — Table S1: Accession numbers of samples used for phylogenetic analyses of Ranunculus. [file ECE3-15-e72191-s001.docx]

**Table S1** Accession numbers of samples used for phylogenetic analyses of *Ranunculus*.

| Taxon | Voucher | ITS | *matK* |
| --- | --- | --- | --- |
| Clade IX |  |  |  |
| *Ranunculus afghanicus* P958 | Kononov A.A. | PQ686768 | PQ723649 |
| *Ranunculus alajensis* P943 | Erst A.S., Bovoev M.T., Boboev K.M. | PQ686760 | PQ723641 |
| *Ranunculus albertii* P953 | Kochkareva T., Riabkova | PQ686763 | PQ723644 |
| *Ranunculus alpigenus* P954 | Sidorenko G.T. | PQ686764 | PQ723645 |
| *Ranunculus arvensis* P938 | Erst A.S., Bobokolonov K.A. | PQ686755 | PQ723636 |
| *Ranunculus aureopetalus* P955 |  | PQ686765 | PQ723646 |
| *Ranunculus baldshuanicus* P939 | Erst A.S., Bobokolonov K.A. | PQ686756 | PQ723637 |
| *Ranunculus boboevii* | Erst A.S., Bovoev M.T., Boboev K.M. | PQ686779 | NA |
| *Ranunculus botscantzevii* P956 | Karimova, Devochkina | PQ686766 | PQ723647 |
| *Ranunculus czimganicus* P959 | Bahmush Yu. | PQ686769 | PQ723650 |
| *Ranunculus komarovii* P964 | Kochkareva, Sharypova | PQ686774 | PQ723655 |
| *Ranunculus laetus* P940 | Erst A.S., Bovoev M.T. | PQ686757 | PQ723638 |
| *Ranunculus leptorrhynchus* P941 | Erst A.S., Bovoev M.T. | PQ686758 | PQ723639 |
| *Ranunculus linearilobus* P960 | Kinzikaeva G.K., Bntrindinova L. | PQ686770 | PQ723651 |
| *Ranunculus mogoltavicus* P961 | Plehanov Yu.S., Peczesky | PQ686771 | PQ723652 |
| *Ranunculus nurekensis* P957 | Chukavin A.G., Bachlug, Patashev | PQ686767 | PQ723648 |
| *Ranunculus oligophyllus* P952 | Kamelin R. | PQ686762 | PQ723643 |
| *Ranunculus oxyspermus* P962 | Sidorchenko | PQ686772 | PQ723653 |
| *Ranunculus paucidentatus* P944 | Erst A.S., Bovoev M.T., Boboev K.M. | PQ686761 | PQ723642 |
| *Ranunculus pinnatisectus* P963 | Kamelin R. | PQ686773 | PQ723654 |
| *Ranunculus rubrocalyx* P970 | Erst A.S., Bovoev M.T., Bobokolonov K.A. | PQ686778 | PQ723659 |
| *Ranunculus rufosepalus* P966 | Sidorenko G.T/ | NA | PQ723660 |
| *Ranunculus severzovii* P967 | Chukavina, Chevtaeva, 67215 | PQ686775 | PQ723656 |
| *Ranunculus tenuilobus* P942 | Erst A.S., Bovoev M.T. | PQ686759 | PQ723640 |
| *Ranunculus transalaicus* P968 | Strizova T. | PQ686776 | PQ723657 |
| *Ranunculus turkestanicus* P969 | Erst A.S., Bovoev M.T., Bobokolonov K.A. | PQ686777 | PQ723658 |
| *Ranunculus aduncus* |  | AY680088 | AY954143 |
| *Ranunculus afghanicus* |  | HQ338297 | HM565146 |
| *Ranunculus alaiensis* |  | MT271830 | NA |
| *Ranunculus alpigenus* |  | OM283824 | NA |
| *Ranunculus amblyolobus* |  | HQ338299 | HM565148 |
| *Ranunculus ampelophyllus* |  | FM242842 | FM242778 |
| *Ranunculus apenninus* |  | AY680091 | AY954150 |
| *Ranunculus argyreus* |  | FM242844 | FM242780 |
| *Ranunculus asiaticus* |  | GU257963 | GU257985 |
| *Ranunculus aucheri* |  | HQ338301 | HQ338379 |
| *Ranunculus botschantzevii* |  | MW540744 | MW748677 |
| *Ranunculus brachylobus* |  | HQ338302 | HQ338347 |
| *Ranunculus breyninus* |  | AY680116 | AY954172 |
| *Ranunculus buhsei* |  | FM242860 | FM242796 |
| *Ranunculus bullatus* |  | AY680114 | AY954161 |
| *Ranunculus cappadocicus* |  | AY680117 | AY954173 |
| *Ranunculus carinthiacus* |  | AY680093 | AY954145 |
| *Ranunculus carpaticus* |  | AY680096 | AY954154 |
| *Ranunculus cicutarius* |  | AY680103 | AY954167 |
| *Ranunculus convexiusculus* |  | MW540743 | MW748676 |
| *Ranunculus cortusifolius* |  | AY680101 | AY954160 |
| *Ranunculus creticus* |  | AY954239 | AY954163 |
| *Ranunculus cupreus* |  | AY954240 | AY954164 |
| *Ranunculus damascenus* |  | HQ338309 | HM565153 |
| *Ranunculus elbrusensis* |  | HQ338311 | HQ338352 |
| *Ranunculus garganicus* |  | AY680107 | AY954165 |
| *Ranunculus gouanii* |  | AY680098 | AY954151 |
| *Ranunculus gracilis* |  | AY680120 | AY954171 |
| *Ranunculus gregarius* |  | AY680100 | AY954159 |
| *Ranunculus heterorrhizus* |  | HQ338317 | HM565156 |
| *Ranunculus hierosolymitanus* |  | HQ338318 | HQ338354 |
| *Ranunculus illyricus* |  | AY680119 | AY954162 |
| *Ranunculus leptorrhynchus* |  | HQ338323 | HQ338358 |
| *Ranunculus macropodoides* |  | HQ338326 | HQ338360 |
| *Ranunculus macrorrhynchus* |  | HQ338327 | HM565160 |
| *Ranunculus makaluensis* |  | HQ338329 | HM565161 |
| *Ranunculus marschlinsii* |  | AY680089 | AY954147 |
| *Ranunculus millefoliatus* |  | AY680108 | AY954166 |
| *Ranunculus montanus* |  | AY680094 | AY954149 |
| *Ranunculus ollissiponensis* |  | AY680109 | AY954157 |
| *Ranunculus oxyspermus* |  | FM242863 | FM242799 |
| *Ranunculus paludosus* |  | AY680102 | AY954155 |
| *Ranunculus papyrocarpus* |  | GU257968 | GU257990 |
| *Ranunculus pedatus* |  | MT271837 | NA |
| *Ranunculus platyspermus* |  | MT271838 | NA |
| *Ranunculus pollinensis* |  | AY680097 | AY954152 |
| *Ranunculus pseudomillefoliatus* |  | AY680110 | AY954156 |
| *Ranunculus pseudomontanus* |  | AY680090 | AY954146 |
| *Ranunculus psilostachys* |  | AY680106 | AY954170 |
| *Ranunculus pskemensis* |  | MT271839 | NA |
| *Ranunculus regelianus* |  | HQ338338 | HQ338366 |
| *Ranunculus rumelicus* |  | AY680104 | AY954168 |
| *Ranunculus sartorianus* |  | AY680095 | AY954148 |
| *Ranunculus spicatus* |  | AY954244 | AY954158 |
| *Ranunculus sprunerianus* |  | AY680105 | AY954169 |
| *Ranunculus talassicus* |  | MW540748 | MW748680 |
| *Ranunculus termei* |  | HQ338346 | HQ338372 |
| *Ranunculus tojibaevii* |  | MW540745 | MW748678 |
| *Ranunculus venetus* |  | AY680087 | AY954144 |
| *Ranunculus villarsii* |  | AY680099 | AY954153 |
| Clade VIII |  |  |  |
| *Ranunculus acriformis* |  | HQ338296 | HQ338378 |
| *Ranunculus bonariensis* |  | AY680183 | GU257986 |
| *Ranunculus recurvatus* |  | AY680118 | AY954175 |
| *Ranunculus trilobus* |  | AY680149 | AY954176 |
| Clade VII |  |  |  |
| *Ranunculus arvensis* |  | HQ650550 | HQ650551 |
| *Ranunculus brutius* |  | HQ338304 | HQ338348 |
| *Ranunculus caucasicus* |  | AY680178 | AY954192 |
| Clade VI |  |  |  |
| *Ranunculus cassius* |  | FM242848 | FM242784 |
| *Ranunculus glabriusculus* |  | FM242812 | FM242748 |
| *Ranunculus pinardi* |  | GU257970 | GU257992 |
| Clade V |  |  |  |
| *Ranunculus alismifolius* |  | HQ338298 | HM565147 |
| *Ranunculus hydrophilus* |  | AY680181 | HM565157 |
| *Ranunculus volkensii* |  | EU288424 | EU288396 |
| Clade IV |  |  |  |
| *Ranunculus abortivus* |  | AY680048 | AY954126 |
| *Ranunculus inamoenus* |  | FM242851 | FM242787 |
| *Ranunculus sulphureus* |  | FM242816 | FM242752 |
| Clade III |  |  |  |
| *Ranunculus apiifolius* |  | AY680092 | AY954140 |
| *Ranunculus aquatilis* |  | FM242843 | FM242779 |
| *Ranunculus collinus* |  | AY680059 | AY954137 |
| *Ranunculus hyperboreus* |  | AY680065 | AY954135 |
| *Ranunculus insignis* |  | AF323306 | AY954141 |
| Clade II |  |  |  |
| *Ranunculus aconitifolius* |  | AY680081 | AY954217 |
| *Ranunculus bilobus* |  | AY680077 | AY954220 |
| *Ranunculus crenatus* |  | AY680086 | AY954228 |
| *Ranunculus pyrenaeus* |  | AY680074 | AY954225 |
| Clade I |  |  |  |
| *Ranunculus brevifolius* |  | AY680187 | AY954212 |
| *Ranunculus hybridus* |  | AY680189 | AY954211 |
| Outgroup |  |  |  |
| *Ceratocephala falcata* |  | AY680191 | GU257996 |
| *Krapfia clypeata* |  | EU053930 | DQ490058 |
| *Myosurus minimus* |  | AJ347913 | FJ626502 |
