# Supplementary material for: Taxonomic Diversity of Ranunculus Section Ranunculastrum (Ranunculaceae) in Tajikistan, With an Identification Key and a New Species Based in Part on Molecular Phylogenetic Evidence
Source: Ecol Evol. 2025 Sep 22;15(9):e72191. doi: 10.1002/ece3.72191 (PMC12453610; doi:10.1002/ece3.72191)
Supplement: Supplementary file 2 — Table S2: Primers used for amplification and sequencing in this study. [file ECE3-15-e72191-s002.docx]

**Table S2** Primers used for amplification and sequencing in this study.

| Locus | Primer | Sequence (5’-3’) | Reference |
| --- | --- | --- | --- |
| ITS | ITS-1 | CCTTATCATTTAGAGGAAGGAG | White et al. (1990) |
|  | ITS-4 | TCCTCCGCTATTGATATGC | White et al. (1990) |
| *matK* | AF2 | CTTTCAGGARTACATTTATGC | Wang et al. (2007) |
|  | 8R2 | ACGWGCCAAAGTTCTAGCAC | Wang et al. (2007) |
|  | mF2 | AAACAATCTTMTCATTTACG | Wang et al. (2007) |
|  | mR2 | AARGGATCCTTGAACAMCCA | Wang et al. (2007) |

**Reference**

White T. J., T. Bruns, S. Lee, et al. 1990. “Amplification and direct sequencing of fungal ribosomal RNA genes for phylogenetics” In PCR protocols: a guide to methods and applications, edit by Innis M. A., D. H. Gelfand, J. J. Sninsky, et al. 315–322. New York: Academic Press.

Wang W., Z. D. Chen, Y. Liu, et al. 2007. “Phylogenetic and Biogeographic Diversification of Berberidaceae in the Northern Hemisphere”. *Systematic Botany*, no. 32: 731–742.
